# Conserved Peptide Upstream Open Reading Frames are Associated with Regulatory Genes in Angiosperms

**DOI:** 10.3389/fpls.2012.00191

**Published:** 2012-08-24

**Authors:** Richard A. Jorgensen, Ana E. Dorantes-Acosta

**Affiliations:** ^1^Laboratorio Nacional de Genómica para la Biodiversidad, Centro de Investigación y Estudios Avanzados del Instituto Politécnico NacionalIrapuato, Guanajuato, México; ^2^Instituto de Biotecnología y Ecología Aplicada, Universidad VeracruzanaXalapa, Veracruz, México

**Keywords:** translational control, gene regulation, peptoswitch, sucrose signaling, polyamine signaling, threhalose signaling, regulatory networks, dicistronic transcripts

## Abstract

Upstream open reading frames (uORFs) are common in eukaryotic transcripts, but those that encode conserved peptides occur in less than 1% of transcripts. The peptides encoded by three plant conserved peptide uORF (CPuORF) families are known to control translation of the downstream ORF in response to a small signal molecule (sucrose, polyamines, and phosphocholine). In flowering plants, transcription factors are statistically over-represented among genes that possess CPuORFs, and in general it appeared that many CPuORF genes also had other regulatory functions, though the significance of this suggestion was uncertain (Hayden and Jorgensen, 2007). Five years later the literature provides much more information on the functions of many CPuORF genes. Here we reassess the functions of 27 known CPuORF gene families and find that 22 of these families play a variety of different regulatory roles, from transcriptional control to protein turnover, and from small signal molecules to signal transduction kinases. Clearly then, there is indeed a strong association of CPuORFs with regulatory genes. In addition, 16 of these families play key roles in a variety of different biological processes. Most strikingly, the core sucrose response network includes three different CPuORFs, creating the potential for sophisticated balancing of the network in response to three different molecular inputs. We propose that the function of most CPuORFs is to modulate translation of a downstream major ORF (mORF) in response to a signal molecule recognized by the conserved peptide and that because the mORFs of CPuORF genes generally encode regulatory proteins, many of them centrally important in the biology of plants, CPuORFs play key roles in balancing such regulatory networks.

## Introduction

Upstream open reading frames (uORFs) occur in 20–50% of eukaryotic transcripts (Kochetov, 2008). However, those encoding conserved peptides uORFs (CPuORFs) are relatively rare, occurring in less than 1% of angiosperm (Hayden and Jorgensen, 2007; Tran et al., 2008), dipteran (Hayden and Bosco, 2008), and mammalian (Crowe et al., 2006) transcripts. The function of CPuORFs has been studied in only a few cases, but in each case the CPuORF has been shown to control translation of a downstream ORF that is modulated by a small signal molecule. Three such examples are known in plants: *S*-adenosylmethionine decarboxylase, which is translationally regulated by polyamines (Ivanov et al., 2010), S1-group bZIP transcription factors, translationally regulated by sucrose (Rahmani et al., 2009), and phosphoethanolamine *N*-methyltransferase, translationally regulated by phosphocholine (Alatorre-Cobos et al., 2012).

Twenty-six different CPuORFs were identified in plants by comparison of rice and *Arabidopsis* full-length cDNAs (Hayden and Jorgensen, 2007). Many CPuORF genes appeared to have regulatory functions, and transcription factors were statistically over-represented among these genes. Here we reevaluate and extend the association of CPuORF genes with regulatory functions through detailed analysis of the literature and consider the hypothesis that the function of the CPuORFs is to “fine tune” translation of downstream ORFs according to a signal to which the CPuORF peptide responds and further that CPuORF genes encode key regulatory proteins that mediate important responses to the environment as well as in growth and development. CPuORF peptides may be thought of as “peptoswitches,” by analogy to riboswitches, which are RNA structures that bind small molecules and thereby attenuate transcription and translation in prokaryotes and regulate splicing in eukaryotes according to the concentration of the small molecule.

## Three Classes of Candidate CPuORFs

Hayden and Jorgensen (2007) developed a program called *uORFinder* to pair the two most similar full-length cDNA sequences between two distantly related genomes and to identify candidate CPuORFs upstream of the major ORF (mORF). By comparison of *Arabidopsis* and rice cDNAs, *uORFinder* detected two classes of candidate CPuORF-carrying transcripts: (1) alternatively spliced transcripts that encode ORFs with alternate N-termini, where one alternative transcript encodes a candidate CPuORF that in the other alternative transcript is fused to the major downstream ORF (mORF), and (2) transcripts that are not alternatively spliced. The latter were considered to represent putatively “true” CPuORFs. We suggest that a third class also exists, namely, dicistronic transcripts, which encode two trans-acting proteins, and that this class should be considered separately. Although dicistronic transcripts are relatively rare in plants, some other taxa, such as dipterans, possess hundreds of such genes (Lin et al., 2011). Thus, while dicistronic genes are at most a minor “contaminant” in CPuORF detection in plants, they are expected to be a major class in some other taxa. (Note: We do not exclude the possibility that the uORF of dicistronic transcripts might function also in regulating translation of the downstream ORF).

By reanalysis of more recently available *Arabidopsis* and rice cDNAs with *uORFinder*, we have also identified three new “true” CPuORF families (HG27–29) and one new discistronic transcript (HG30). The putative CPuORF of HG30 encodes a cdc26 homolog. Among the original 26 angiosperm CPuORF families of Hayden and Jorgensen (2007), HG8’s CPuORF peptide is the only one that encodes a known domain, a CHCH domain that appears to belong to the twin CX_9_C class of CHCH proteins (Cavallero, 2010). Cavallero (2010) suggested that twin CX_9_C proteins play a scaffolding role in mitochondrial structure and function, although HG8’s CPuORF is a member of twin CX_9_C cluster 21, which lacks any known function. HG8’s CX_9_C protein is conserved among plants, animals, and fungi, yet is associated with different mORFs in different taxa, and even exists free of any mORF in some taxa (Hayden and Jorgensen, 2007). Thus, we suggest that HG8 should be considered as a probable dicistronic transcript.

All detected CPuORF families and dicistronic genes are shown in Table 1.

**Table 1:** **CPuORF genes: mORF molecular function and biological role**.

Homology group	*Arabidopsis* locus ID	*Arabidopsis* gene name	mORF: known or inferred molecular function/domain	Known or inferred biological role	Comments	Citations
**CONTROL OF TRANSCRIPTION**						
HG1	At4g34590	*bZIP11 (GBF6)*	bZIP transcription factor	Transcriptional control Controls amino acid metabolism in response to sucrose status	S1-group bZIPs Sucrose modulates translation via CPuORF	Rahmani et al. (2009)
	At2g18160	*bZIP2 (GBF5)*	
	At3g62420	*bZIP53*				
	At5g49450	*bZIP1*	
	At1g75390	*bZIP44*	
HG2	At2g27230	*LHW (BHLH156*	bHLH transcription factor	Transcriptional control Root stele determination and lateral root initiation	SACL3 (HG15) binds LHW	Parizot et al. (2008), Ohashi-Ito and Bergmann (2007)
	At2g31280	*BHLH155*			
	At1g06150	*EMB1444*	
HG4	At5g52550		MADS-box, MIKC^C^ type transcription factor	Transcriptional control	Annotation based on rice ortholog Os02g01360 (OsMADS60)	Arora et al. (2007), Gramzow and Theissen (2010)
	At4g25670	
	At4g25690	
HG14	At3g01470	*HD-ZIP1 (AtHB-1)*	HD-Zip class I transcription factor	Transcriptional control Photosynthetic light acclimation	Highly connected hub in light acclimation	Yao et al. (2011)				
HG15	At5g64340 At5g09460 At1g29950 At5g50010	*SAC51 (BHLH142)* *SACL1 (BHLH143)* *SACL3 (BHLH144)* *SACL2 (BHLH145)*	bHLH transcription factor	Transcriptional control Vascular (xylem) development	Thermospermine activates translation of SAC51; SACL3 binds LHW (HG2)	Imai et al. (2006), Kakehi et al. (2008), Takahashi and Kakehi (2009)
HG18	At4g36990	*TBF1 (HsfB1; HSF4)*	HSF-like transcription factor	Transcriptional control Growth-to-defense transition	Central regulator in pathogen response	Pajerowska-Mukhtar et al. (2012)				
HG21	At1g25470	*ERF116 (CRF12)*	AP2 transcription factor; cytokinin response factor	Transcriptional control	Cytokinin responsive	Rashotte and Goertzen (2010)
	At1g68550	*ERF118 (CRF10)*	
**CONTROL OF TRANSLATION AND PROTEIN DEGRADATION**						
HG7	At1g36730	*eIF5-1*	Translation initiation factor eIF5	Regulates general translation in response to amino acid starvation	Start codon selection; GDI and GAP activity	Jennings and Pavitt (2010)
HG22	At1g78880		Ubiquitin-specific protease family C19-related	Regulation of ubiquitin-dependent protein degradation		Reyes-Turcu et al. (2009)
	At1g16860	
HG26	At5g05280	*ATL73*	RING finger protein; E3 ubiquitin ligase	Mediator of specific protein degradation		Aguilar-Hernández et al. (2011)
	At3g10910	*ATL72*	
HG28	At1g67480		F-box/kelch-repeat protein	F-box determines substrate recognition by SCF ubiquitin ligases	Similar to SKIP6 (SKP1-interacting partner 6)	Xu et al. (2009)
**SIGNALING – SMALL MOLECULE SIGNALS**						
HG3	At3g02470	*AdoMetDC1*	*S*-adenosylmethionine decarboxylase	Polyamine biosynthesis	Regulates growth, development and stress responses	Ivanov et al. (2010)
	At5g15950	*AdoMetDC2*	
	At3g25570	*AdoMetDC3*	
HG6	At2g43020	*PAO2*	Polyamine oxidase	Catabolizes spermidine, norspermine, thermospermine	Growth, development and stress responses	Takahashi et al. (2010), Fincato et al. (2011)
	At3g59050	*PAO3*	
HG11	At4g12430 At4g22590	*TPP5 (TPPF)* *TPP6 (TPPG)*	Trehalose-6-phosphate phosphatase	T6P inhibits SNrK1 indicator of sucrose status, regulates sucrose utilization and carbon partitioning	TPP6 lacks synthase domain	Ponnu et al. (2011), Ma et al. (2011), Valluru and Van den Ende (2011)
HG13	At3g18000	*XPL1 (NMT1)*	Phosphoethanolamine *N*-methyltransferase	Phosphatidic acid and phosphocholine biosynthesis; root development	Phosphocholine regulates translation of XPL1 via CPuORF	Alatorre-Cobos et al. (2012)
	At1g48600	*NMT2*	
	At1g73600	*NMT3*	
**SIGNALING – SIGNAL TRANSDUCTION KINASES**						
HG10	At4g19110		MAP kinase, PPC family 4.5.1	Signal transduction; *Chlamydomonas* ortholog LF4 regulates flagellar length	Pollen and sperm cell-specific expression	Berman et al. (2003)
	At5g45430	
HG16	At3g51630	*WNK5 (ZIK1)*	MAP kinase, PPC family 4.1.5	Signal transduction		
HG23	At1g64630	*WNK8* *(ZIK6)*	MAP kinase, PPC family 4.1.5	Signal transduction promotes flowering	Phosphorylates EDM2 and V-ATPase	Tsuchiya and Eulgem (2010)
	At5g41990	*WNK10 (ZIK10)*	
HG25	At5g60550	*GRIK1 (SNAK2)*	Calcium response protein kinase	Ca++/CaM−dependent signal transduction; sugar signaling	Phosphorylates SNrK1	Shen and Hanley-Bowdoin (2006)
	At3g45240	*GRIK2 (SNAK1)*	
HG27	At4g30960	*CIPK6 (SIP2)*	CBL-interacting protein kinase	Signal transduction salt and drought tolerance	Activates K^+^ transporter AKT2	Tsou et al. (2012), Dreyer and Uozumi (2011)
**OTHER REGULATORY FUNCTION**						
HG5	At5g61230	*ANK6*	Ankyrin repeats – protein–protein interaction	Male-female gamete recognition	Interacts with mitochondrial sigma factor SIG5	Yu et al. (2010)
	At5g07840	
HG29	At2g22500	*DIC1*	Dicarboxylate carrier protein dicarboxylic acid transporter	Redox connection between mitochondria and cytosol		Palmieri et al. (2008)
**OTHER FUNCTION OR UNKNOWN FUNCTION**						
HG9	At5g64550		Cysteine-rich protein: CX_4/7_CX_10_CX_2_HX_5_ tandem repeats	Unknown	Novel zinc finger?
	At5g09670	
	At1g64140	
HG12	At1g23150		Unknown	Unknown		
	At1g70780	
HG19	At5g53590	*HIRP1*	Histidine-rich SAUR-like protein	Unknown	Metal binding: metal homeostasis or tolerance?	Hara et al. (2010)
HG20	At2g37480		Unknown	Unknown		
	At3g53670	
HG24	At3g22970		DUF506; PD-(D/E)XK nuclease	Unknown		Knizewski et al. (2007)
	At4g14620	
**PUTATIVE DICISTRONIC TRANSCRIPTS (NOT “TRUE” CPuORFs?)**						
HG8	At3g12010		TGF-beta superfamily; Mic-1 (GDF15) putative ortholog	Cell cycle regulation, S phase arrest, and starvation for DNA precursors (in animals)	CPuORF conserved across eucaryotes; “free” of mORF in many taxa	Agarwal et al. (2006), Cavallero (2010)
HG30	At2g11890		Adenylate cyclase	cAMP biosynthesis	CPuORF is cdc26	

## CPuORFs are Associated with Regulatory Genes in Angiosperms

Hayden and Jorgensen (2007) reported that transcription factors were significantly over-represented among CPuORF families and noted also that most CPuORFs were associated with regulatory genes. Since that time, a great deal of new information has been published in the literature regarding functions in *Arabidopsis* of the mORFs associated with many of the identified CPuORF genes. By means of a comprehensive literature and database analysis, we have been able to identify known or inferred functions for the mORFs encoded by 22 of the 27 putatively “true” CPuORF families, summarized in Table 1. Remarkably, all 22 of these mORFs with a known or inferred function appear to play a regulatory role:

(1) Seven (26%) CPuORF families encode a variety of different types of transcription factor (Table 1), including bZIP (HG1), bHLH (HG2 and HG15), MADS (HG4), HD-ZIP (HG14), AP2/ERF (HG21), and an HSF-like activator (HG18). [Note: One of these (HG4) was not identified as a transcription factor by Hayden and Jorgensen (2007) because it had not been annotated as such in public database; however, the rice member of this family has been reported to be a MADS transcription factor by Arora et al. (2007) and Gramzow and Theissen (2010).
(2) Nine (33%) CPuORF families are involved in signaling processes (Table 1): (a) Five of these families encode signal transduction protein kinases, including three MAP kinases (HG10, HG16, and HG23), a calcium response kinase (HG25), and a CBL-interacting protein kinase (HG27). (b) Four families are involved in the biosynthesis or catabolism of small molecule signals, including polyamines (HG3 and HG6), trehalose (HG11), and phosphocholine (HG13).
(3) Four (15%) CPuORF families are involved in control of translation or protein degradation (Table 1). One family encodes translation initiation factor eIF5, whereas three other CPuORF families are predicted to play roles in the regulation of protein degradation: The HG22 mORF encodes a ubiquitin-specific protease, proteins which are implicated in regulation of ubiquitin-dependent protein degradation (Reyes-Turcu et al., 2009). HG26’s mORF encodes an E3 ubiquitin ligase, a protein that mediates degradation of a specific target protein(s) (Aguilar-Hernández et al., 2011). The mORF of HG28 encodes an F-box domain protein; F-boxes function in substrate recognition by SCF ubiquitin ligases, determining specificity.
(4) CPuORF family HG5 encodes ANK6, an ankyrin repeat protein that is proposed to play a central role in male-female gamete recognition by regulating mitochondrial gene expression (Yu et al., 2010). It interacts in mitochondria with the non-conserved N-terminal region of SIG5, a mitochondrial sigma factor. By analogy to bacterial sigma factor 70, Yu et al. (2010) propose that by interacting with the inhibitory N-terminal sequence of SIG5, ANK6 uncovers SIG5’s C-terminal domain which then binds both to the mitochondrial RNA polymerase core to form a holoenzyme and to a mitochondrial promoter, initiating transcription of gamete-specific genes necessary for gamete recognition.
(5) Finally, CPuORF family HG29 encodes DIC1, a mitochondrial dicarboxylate carrier which imports dicarboxylates into mitochondria to replenish the TCA cycle and is proposed to function as a malate/oxaloacetate shuttle providing other cellular compartments with reducing equivalents. When this shuttle is coupled with the chloroplastic malate valve, the plant acquires the ability to balance cellular energy supply and control redox poise (Palmieri et al., 2008).

Of the five genes of unknown function (19%), three have domains that are not sufficient to suggest whether these proteins perform a regulatory function:

(1) HG9 encodes a cysteine-rich protein in which we detected CX_4/7_CX_10_CX_2_HX_5_ tandem repeats that to our knowledge have never been described in the literature or databases. Conceivably, it could be a novel zinc finger domain, but its function is unknown.
(2) HG19 encodes a histidine-rich SAUR-like protein that binds metal ions *in vitro* (Hara et al., 2010). Conceivably, it could be involved in metal tolerance or homeostasis, but its function is not known.
(3) HG24 encodes a domain of unknown function, DUF506 which has been shown be a member of the PD-(D/E)XK nuclease superfamily that has many functions, including restriction endonucleases, transposases, and roles in DNA degradation, recombination, and repair, and RNA processing (Knizewski et al., 2007). The DUF506 family has 18 members in rice and 23 in *Arabidopsis*, but none have any known function.

**Figure 1:**
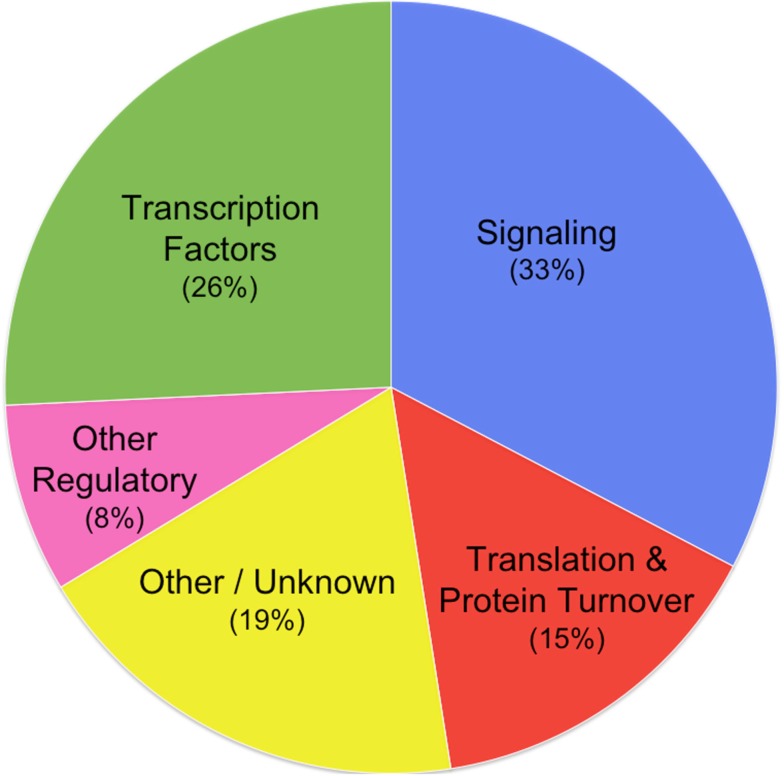
**Relative frequency of functional classes**.

The distribution of functional classes is diagrammed in Figure 1.

Although the two putative dicistronic families, HG8 and HG30, have not been included in this summary, our conclusion would not be weakened by their inclusion because both appear to encode regulatory genes. The HG8 mORF encodes a member of the TGF-beta superfamily, an ortholog of the Mic-1/GDF15 gene, which is involved in cell cycle regulation in response to starvation for DNA precursors in animals. HG30’s mORF encodes adenylate cyclase, which is involved in cyclic AMP biosynthesis; cyclic AMP is a small signaling molecule. Thus, inclusion of HG8 and HG30 would only reinforce our conclusion that CPuORFs are associated with regulatory genes.

It is not possible to calculate the statistical significance of the conclusion that CPuORFs are associated with regulatory genes because the genome has not been annotated at the same level of detail as we have re-annotated CPuORF genes. However, with the exception of small signal molecules, biosynthetic, and other “housekeeping” enzymes responsible for primary and secondary metabolism are strikingly absent, such as biosynthesis of carbohydrates, amino acids, fatty acids, nucleotides, enzyme cofactors, sterols, chlorophylls, carotenoids, terpenoids, alkaloids, flavonoids, isoflavonoids, and anthocyanins, as well as polymers such as cellulose, polysaccharides, waxes, lignins, and tannins. Even leaving out small molecule signal genes, the association of CPuORFs with regulatory genes (e.g., transcription factors and signal transduction kinases comprise 12 of the 27 CPuORF families) is very striking.

### Functions associated with genes encoding alternative N-termini due to alternative splicing

Some alternatively spliced genes that encode proteins with different N-termini due to fusion of the uORF and mORF of one transcript into a single ORF in the alternatively spliced transcript, i.e., the N-terminus of the second alternative transcript is the same as the N-terminus of the uORF of the first transcript. Hayden and Jorgensen (2007) did not report the identities of genes in the alternative N-termini (aNT) class, so we repeated the analysis of *Arabidopsis* and rice full-length cDNAs with *uORFinder* and detected 25 cases of N-terminal alternative splicing (Table A1 in Appendix). (Note: Based on alternative transcripts now available in public databases, we have reclassified family HG17 as aNT family aNT25.) Alternative transcripts encoding similar proteins with different N-termini were produced by several different types of alternative splicing: intron retention, alternative acceptor site, and alternative splice site selection.

The spectrum of functions of the 25 aNT genes (Table A1 in Appendix) is strikingly different than the spectrum of functions found in CPuORF genes (Table 1). Although a number of these genes play regulatory roles, what is most striking is the abundance of proteins associated with membranes or organelles: nine are localized to the plasma membrane and endomembrane system seven in chloroplasts (four of these in chloroplast membranes), six in the nucleus, one to the cytoskeleton, one to the cytosol, and one unknown location. Alternative splicing to produce aNT has been shown in some cases to be responsible for alternative localization of proteins to different compartments in plant cells (Silva-Filho, 2003), and this may also be the case with the alternatively spliced, aNT transcripts identified by *uORFinder*. These aNT genes function in diverse processes, including glycolysis, cell wall biogenesis, membrane fusion, microtubule organization, spliceosome assembly, apoptosis, light harvesting, membrane targeting and transport, and biosynthesis of chlorophyll and phylloquinone in the chloroplast. The striking difference in the spectrum of functions for the aNT class as compared to the spectrum of functions identified for the CPuORF class lends support to the observation that CPuORFs are associated with regulatory genes in plants.

## Diverse Types of Regulatory Processes are Associated with CPuORF Genes

Extensive review of the experimental research literature showed that 16 CPuORF families are involved in key regulatory processes in growth, development, and physiology (Table 1), as follows.

### The sucrose status response network involves five CPuORF families

**Figure 2:**
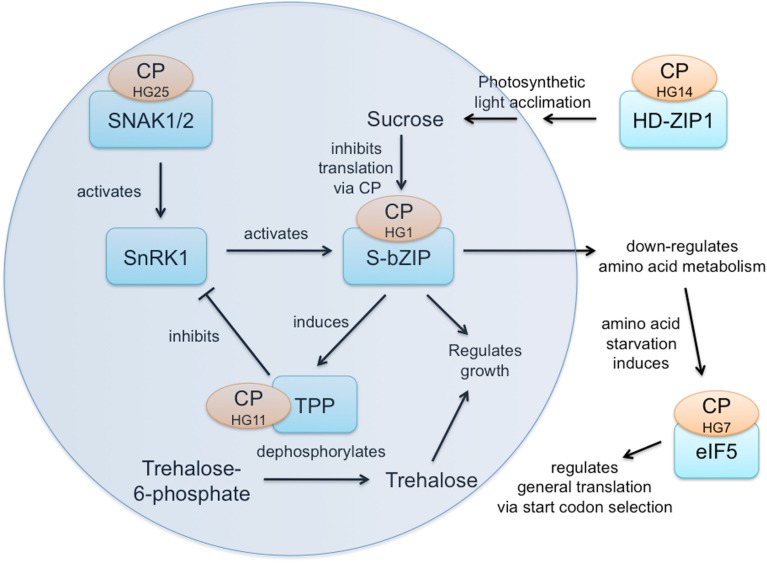
**Sucrose status response network**.

Sucrose is a signal molecule in plants and has been shown to modulate translation of the S1-group of bZIP transcription factors in order to adjust metabolism to altered sucrose status (Rahmani et al., 2009). The core SnRK1/T6P/S-bZIP sucrose status response network (Figure 2) includes three CPuORF families:

(1) SnRK1 activates SNAK1/2 (Shen and Hanley-Bowdoin, 2006), which have the HG25 CPuORF.
(2) SnRK1 acts through S1-bZIP and the other S1-group bZIP transcription factors (HG1 family) whose translation is modulated by sucrose via the HG1 CPuORF (Rahmani et al., 2009).
(3) S1-bZIP induces transcription of TPP5 and TPP6 (trehalose-6-phosphate phosphatases) whose translation may be modulated via the CPuORF of HG11. Trehalose-6-phosphate (T6P) is known to inhibit SnRK1 (Zhang et al., 2009).

Homeobox leucine zipper transcription factor HD-ZIP1 (HG14) is a highly connected central hub in the long-term photosynthetic light acclimation response whose expression is induced by a signal of the oxidized state of the plastoquinone pool (Yao et al., 2011), thereby influencing sucrose levels and the sucrose status response network. In addition, the S1-bZIP transcription factors regulate growth, in part by down-regulating amino acid metabolism in response to sucrose acting through CPuORF family HG1. Amino acid starvation induces translation initiation factor eIF5 (HG7), which regulates general translation by altering start codon selection (Jennings and Pavitt, 2010), potentially influencing translation of many transcripts possessing upstream ATGs. Thus, five CPuORF families participate in, influence, or respond to this energy sensing network, diagrammed in Figure 2. Only the HG1 CPuORF has an experimentally determined function in regulating mORF translation. Clearly, it would be very interesting to determine the functions of the other four CPuORF families and the nature of any signals to which they might respond.

### Three CPuORF families are involved in polyamine metabolism or action

Polyamines are small signal molecules that play important roles in both vascular development and abiotic stress tolerance (Takahashi and Kakehi, 2009; Alcázar et al., 2010; Vera-Sirera et al., 2010). Three CPuORF families involve polyamines:

(1) HG3 encodes *S*-adenosylmethionine decarboxylase, which is required for polyamine biosynthesis (Ivanov et al., 2010).
(2) HG6 encodes polyamine oxidases that catabolize spermidine, norspermidine, and thermospermine (Takahashi et al., 2010; Fincato et al., 2011).
(3) Mutation of the HG15 CPuORF suppresses thermospermine synthase mutations, and thermospermine is thought to regulate translation of SAC51 via the HG15 CPuORF (Takahashi and Kakehi, 2009; Vera-Sirera et al., 2010). SAC51 encodes a bHLH transcription factor necessary for xylem development (Imai et al., 2006).

### Other regulators of plant metabolism and responses to the environment

Three additional CPuORF families play key roles in the acclimation of physiology and metabolism to changing conditions:

(1) TBF1 (HG18) encodes a transcription factor that is a “master molecular switch” in the growth-to-defense transition, activating, and repressing more than 1,000 genes that carry a TBF1 binding site in their promoters (Pajerowska-Mukhtar et al., 2012). TBF1 adjusts physiology in response to pathogen attack via its uORF, which is rich in phenylalanine and regulates translation in response phenylalanine starvation. Thus, TBF1 senses metabolic changes during pathogen attack and triggers specific transcriptional reprogramming. However, the phenylalanine richness of this CPuORF does not explain the highly conserved 22 amino acid segment of the CP. The possibility should be considered that this CP recognizes some signal molecule that regulates translation via the interaction.
(2) DIC1 (HG29), as mentioned above, has been proposed to balance cellular energy supply and control redox poise (Palmieri et al., 2008).
(3) CIPK6 (HG27) is a serine/threonine protein kinase whose up-regulation results in enhanced drought and salt tolerance (Tsou et al., 2012).

### Regulation of development

Six other CPuORF families play important roles in the regulation of development:

(1) LONESOME HIGHWAY (LHW; HG2) encodes a bHLH transcription factor necessary for root stele determination and lateral root initiation (Ohashi-Ito and Bergmann, 2007).
(2) As was mentioned in the discussion of polyamines, SAC51 (HG15) encodes a bHLH transcription factor necessary for xylem development (Imai et al., 2006).
(3) ANK6 (HG5), as was mentioned above, is believed to regulate transcription of gamete-specific genes in the mitochondrial genome, thereby controlling male-female gamete recognition (Yu et al., 2010).
(4) XPL1 (HG13) is required for phosphocholine synthesis in plants. Phosphocholine concentration regulates the proliferative capacity of root meristems, the proper elongation of root cells, and the viability of the elongating root epidermal cells. Phosphocholine regulates XPL1 translation via its CPuORF (Alatorre-Cobos et al., 2012).
(5) WNK8 (HG23) acts upstream of EDM2 in an FLC-dependent mechanism that controls the timing of the floral transition and counteracts the promoting effect of EDM2 on this developmental process, i.e., it is a key component of a regulatory module that resembles the autonomous floral promotion pathway (Tsuchiya and Eulgem, 2010).
(6) Family HG10 encodes a MAP kinase that is highly conserved between plants and animals. The HG10 ortholog in *Chlamydomonas*, LF4, regulates flagellar length (Berman et al., 2003). LF4 has no role in assembly, but rather maintains a specific length in assembled flagella, apparently by shortening. Interestingly, the plant HG10 family is expressed specifically in pollen and sperm cells where it may serve a similar function.

### Biological process unknown

Six families whose mORFs encode domains inferred to have a regulatory function have not been associated experimentally with a specific biological process. Neither have the five families whose mORF functions are unknown been associated experimentally with a biological process. Given the important roles of the 16 better-characterized CPuORF families, it is interesting to consider that some of these other 11 families might play similarly important roles in the biology of plants.

## Discussion

### The hypothesis for modulating mORF translation via CPuORFs

The three families whose CPuORFs have been investigated experimentally regulate mORF translation via the CPuORF in response to a small molecule signal: translation of bZIP11 (HG1) is regulated by sucrose via its CPuORF; translation of *S*-adenosyl methionine decarboxylase (HG2) is regulated by polyamines via its CPuORF; and translation of phosphoethanolamine *N*-methyltransferase is regulated by phosphocholine via the HG13 CPuORF. In each case, the CPuORF has been shown to modulate, or “fine tune,” translation of its downstream mORF in response to a particular small molecule signal. Thus, an attractive hypothesis is that other CPuORF families also exhibit small molecule regulation of mORF translation. In other words, CPuORFs act as “peptoswitches,” by analogy to riboswitches which are RNA structures that bind small molecules and attenuate transcription and translation in prokaryotes and regulate splicing in eukaryotes.

Our observation that many CPuORF genes are associated with key regulatory processes suggests an expanded hypothesis, i.e., that CPuORFs play a major role in the biology plants by sensitively modulating translation of key regulatory proteins in response to specific signal molecules, thereby optimizing metabolic, physiological, and developmental networks for particular conditions.

Given the central importance of many CPuORF genes in the biology of plants, we would suggest that analysis of all CPuORF families should be a priority for experimental research designed to ascertain whether such signal molecules exist and regulate mORF translation, and if they do, to determine their molecular natures and whether they fine tune gene expression networks. This is important at two levels. First, in the 16 CPuORF families with known regulatory roles it would be interesting to investigate the potential role of the CPuORF in regulating translation of its downstream mORF, as well as the nature of any signal molecule that might act through the CPuORF. Second, in the 12 families lacking an experimentally determined role in regulation, it could be very interesting to learn the role played in the biology of plants because these genes may also play central roles in the biology of plants. Given the apparently key regulatory roles that many CPuORF genes play, we feel that such experiments should be given a high priority relative to many other genes whose function also remains unknown or poorly understood.

### Selective regulation of translation initiation in plants

Selective translation of mRNAs has been shown to play an important role in some plant acclimation responses. For instance, cellular oxygen deprivation requires plants to acclimatize in order to survive. It has been shown that the conservation of energy and the transition to anaerobic metabolism that occur during such hypoxia stress is coordinated by selective mRNA translation, resulting in the inhibition of translation of ~70% of cellular mRNAs (Branco-Price et al., 2008). CPuORF genes represent a specific class of “selectively” translated genes that are relatively rare. CPuORFs were estimated by Hayden and Jorgensen (2007) to occur in only ~0.5% of *Arabidopsis* genes.

### Further considerations of three CPuORF genes

#### eIF5 (HG7)

The presence of one or more uORFs is a near universal feature of eukaryotic eIF5 mRNAs (Loughran et al., 2011). Experiments in human cells have shown that eIF5 mRNA autoregulates its own translation via inhibitory uORFs. eIF5 protein is known to increase the stringency of start codon selection, whereas at high eIF5 levels it induces translation initiation at AUGs in poor contexts. Because the uORF AUG(s) of eIF5 mRNAs have poor initiation contexts and translation of these uORFs is inhibitory to translation of the eIF5 mORF, high levels of eIF5 reduce translation of the eIF5 mORF, providing negative feedback (Loughran et al., 2011). Given that plant eIF5 mRNAs encode a CPuORF (HG7), we suggest that modulation of eIF5 translation by an unknown signal molecule triggers a general shift in mRNA translation in favor of AUGs in “poor” initiation contexts at the expense of those in “good” contexts, thereby mediating a global change in translation rates of many thousands of mRNAs and shifting the relative abundances of many proteins. Alternatively, the conserved peptide might function by directly interacting with ribosomes or other initiation factors. Distinguishing these possibilities will require direct experimentation.

#### TBF1 (HG18)

TBF1 has been shown to be a key regulator of the growth-to-defense transition (Pajerowska-Mukhtar et al., 2012). The CPuORF of TBF1 is the second of two uORFs in the 5′ UTR that repress TBF1 translation. Both uORF peptide sequences contain four phenylalanine residues, and phenylalanine starvation was shown to alleviate translational repression by the uORFs. Pathogen challenge increases uncharged tRNA^phe^ and phosphorylation of translation initiation factor eIF2alpha and releases the inhibitory effects of the uORFs on TBF1 translation. This is similar to GCN4 in yeast whose translation is derepressed by amino acid starvation via accumulation of uncharged tRNAs that induce phosphorylation of eIF2alpha, allowing translation to reinitiate at the GCN4 start codon. Translational control allows rapid response to pathogen detection, via the rapid, dramatic increase in uncharged tRNA^phe^, by producing TBF1 protein from transcripts already present, thereby activating the immune response (Pajerowska-Mukhtar et al., 2012). An important question that remains about TBF1 is why the peptide sequence of its second uORF is conserved. One possibility is that the peptide interacts with the ribosome to influence whether it is able to reinitiate at the TBF1 start codon, though it should be noted that none of the GCN4 uORFs is conserved. The other possibility that should be considered is that the HG18 conserved peptide interacts with a signal molecule that could influence translation.

#### CIPK6 (HG27)

CIPK6 is a serine/threonine kinase that enhances drought and salt tolerance when upregulated. It appears to do so by modulating the activity of potassium channel AKT2 and mediates AKT2 translocation to the plasma membrane (Held et al., 2011). Phosphorylation and desphosphorylation of AKT2 channels control switching between two gating modes, one mode opening channels at voltage more negative than −100 mV and the other mode being voltage independent. Toggling between modes assists in energizing transmembrane transport processes, predominantly in phloem tissues where K^+^ ions serve as decentralized energy storage (Dreyer and Uozumi, 2011). CIPK6 is only one of many CIPKs in plants, and presumably it regulates AKT2 in response to a specific condition or signal that it transmits to AKT2. Conceivably, translational regulation of CIPK6 could be a key aspect of that response, perhaps leading to more rapid switching of AKT2 channel gating than might be achieved by only a transcriptional response, and possibly modulating the response quantitatively in response to the degree of drought or salt stress.

## Conflict of Interest Statement

The authors declare that the research was conducted in the absence of any commercial or financial relationships that could be construed as a potential conflict of interest.

## Appendix

**Table A1:** **Alternative splicing fuses uORF to mORF**.

Alt NT group	TAIR locus ID	*Arabidopsis* gene name	Inferred molecular function/domain(s)	Inferred biological role/process and cellular location (TAIR)	Alt splice type (*Arabidopsis*)
**LOCALIZED TO PLASMA MEMBRANE AND ENDOMEMBRANE SYSTEM**					
aNT1	At1g02145	*EBS4/ALG12*	alpha-1,6-mannosyltransferase; Alg9-like mannosyltransferase	Protein catabolic process Located in endomembrane system; intrinsic to ER membrane	Intron retention				
aNT3	At1g08350		Endomembrane protein 70 family	Cellular membrane fusion	Intron retention
			Non-aspanin	Integral to membrane, phragmoplast	
aNT5	At1g29890	*REDUCED WALL ACETYLATION (RWA4)*	*O*-acetyltransferase; Cas1p-like	Secondary wall biogenesis Located in Golgi apparatus	Intron retention				
aNT13	At3g45960	*EXPANSIN-LIKE A3 (EXLA3)*	Expansin; endoglucanase	Cell growth, cell wall loosening; located in cytosol, cell wall, membrane, plasmodesma	Intron retention
aNT15	At3g52990	PK90	Pyruvate kinase	Glycolysis Located in cytosol, membrane	Intron retention
aNT18	At5g22140		FAD/NAD(P)-binding oxidoreductase family protein; electron carrier	Oxidation-reduction process Located in plasma membrane, plasmodesma	Alt splice site selection				
aNT19	At5g41610	*CATION/H*+ *EXCHANGER 18 (CHX18)*	Na+/H+ antiporter	Cation transport Located in late endosome	Intron retention				
aNT22	At5g61520	*STP3*	Sugar:hydrogen symporter	Carbohydrate transmembrane transport Integral to membrane	Intron retention
aNT25	At5g01710		Methyltransferase domains: type 11; FkbM	Metabolism Located in endomembrane system	Intron retention (alt NT based on paralog model At5g03190.2)
**LOCATED IN CHLOROPLAST**					
aNT4	At1g23360	*MENG*	2-phytyl-1,4-naphthoquinone methyltransferase	Phylloquinone (vitamin K1) biosynthesis Located in chloroplast	Intron retention (3)
aNT6	At1g61520	*LIGHT HARVESTING COMPLEX A3 (LHCA3)*	PSI type III chlorophyll a/b-binding protein	Photosynthesis, light harvesting; located in chloroplast thylakoid membrane	Intron retention (2)
aNT10	At3g01500	*CARBONIC ANHYDRASE1 (CA1)*	Beta-carbonic anhydrase	Carbon utilization; CO_2_-controlled stomatal movements in guard cells	Alt splice site selection
				Located in chloroplast envelope, stroma, thylakoid membranes	
aNT12	At3g14590	*NTMC2T6.2*	C_2_ calcium-dependent membrane targeting domain	Membrane targeting; located in chloroplast	Alt splice site selection; intron retention
aNT17	At4g35860	*GTP-BINDING 2 (GB2)*	Small GTP-binding protein	Protein transport, signal transduction Located in chloroplast	Intron retention
aNT20	At5g54190	*PROTOCHLOROPHYLLIDE OXIDOREDUCTASE A (PORA)*	Light-dependent NADPH:protochlorophyllide oxidoreductase A	Chlorophyll biosynthesis	Intron retention Located in chloroplast envelope and thylakoid				
aNT21	At5g59950		RNA-binding (RRM/RBD/RNP motifs)	Process unknown Located in chloroplast	Intron retention				
**LOCALIZED TO CYTOSKELETON**					
aNT16	At4g12790	*None*	ATP/GTP-binding site motif A (P-loop)	Paralog QQT1 participates in the organization of microtubules during cell division	uORF of At4g12790 is N-term of paralogs
	At5g22370	*QUATRE-QUART1* (*QQT1)*		Paralog QQT1 localized to mitotic cytoskeleton	QQT1 & QQT2
	At4g21800	*QUATRE-QUART1* (*QQT2)*	
**LOCATED IN NUCLEUS**					
aNT2	At1g04870	*PROTEiN ARGININE METHYLTRANSFERASE 10 (PRMT10)*	histone-arginine *N*-methyltransferase	Histone-arginine methylation Located in nucleus	Alt acceptor site
aNT7	At1g68130	*INDETERMINATE DOMAIN14 (IDD14)/IDD16*	Zinc finger, C2H2-type transcription factor	Regulation of transcription, starch metabolism Located in nucleus	Intron retention
aNT8	At2g35990	*LONELY GUY2 (LOG2)*	Cytokinin riboside 5′-monophosphate phosphoribohydrolase	Cytokinin activating enzyme Located in cytosol and nucleus	Alt splice site selection
aNT9	At2g37340	*RSZ33*	Arginine-serine-rich Zinc knuckle protein	Spliceosome assembly Located in nuclear speck	Alt splice site selection; intron retention				
aNT14	At3g46130	*MYB48*	MYB transcription factor	Regulation of transcription in flavonol biosynthesis; located in nucleus	Alt splice site selection; intron retention
aNT24	At5g64960	*CYCLIN-DEPENDENT KINASE C2 (CDKC2)*	Cyclin-dependent kinase	Involved in development, viral reproduction. colocalizes with microtubule, spliceosomal complex (nucleus)	Intron retention
**LOCATED IN CYTOSOL, OR UNKNOWN LOCATION**					
aNT11	At3g13225		WW/Rsp5/WWP Domain	Protein binding domain Cellular location unknown	Intron retention
aNT23	At5g64830		Programmed cell death protein 2, C-terminal domain	Apoptosis Located in cytoplasm	Alt splice site selection				
